# An Intention-Driven Semi-autonomous Intelligent Robotic System for Drinking

**DOI:** 10.3389/fnbot.2017.00048

**Published:** 2017-09-08

**Authors:** Zhijun Zhang, Yongqian Huang, Siyuan Chen, Jun Qu, Xin Pan, Tianyou Yu, Yuanqing Li

**Affiliations:** ^1^School of Automation Science and Engineering, South China University of Technology, Guangzhou, China

**Keywords:** assistive robot, neural network, semi-autonomous control, brain–machine interface, object recognition and localization

## Abstract

In this study, an intention-driven semi-autonomous intelligent robotic (ID-SIR) system is designed and developed to assist the severely disabled patients to live independently. The system mainly consists of a non-invasive brain–machine interface (BMI) subsystem, a robot manipulator and a visual detection and localization subsystem. Different from most of the existing systems remotely controlled by joystick, head- or eye tracking, the proposed ID-SIR system directly acquires the intention from users’ brain. Compared with the state-of-art system only working for a specific object in a fixed place, the designed ID-SIR system can grasp any desired object in a random place chosen by a user and deliver it to his/her mouth automatically. As one of the main advantages of the ID-SIR system, the patient is only required to send one intention command for one drinking task and the autonomous robot would finish the rest of specific controlling tasks, which greatly eases the burden on patients. Eight healthy subjects attended our experiment, which contained 10 tasks for each subject. In each task, the proposed ID-SIR system delivered the desired beverage container to the mouth of the subject and then put it back to the original position. The mean accuracy of the eight subjects was 97.5%, which demonstrated the effectiveness of the ID-SIR system.

## 1 Introduction

Independent living is essential for the patients with motor deficit due to stroke, spinal cord injures, etc. (Kim et al., 2012; Carlson and del R Millan, 2013; Susko et al., 2016). In order to assist the patients to live independently, intelligent robotics technology is an attractive solution (Hochberg et al., 2012; Wu et al., 2015; He et al., 2016).

With less burden during the task execution period, it is a challenging work to accurately and real-time obtain the intentions of patients, locate the desired object, and efficiently control the robot manipulator to grasp the object and deliver it to the user. Evidently, intention obtaining approach, robot control, and object perception are three key points.

### 1.1 Intention Obtaining Approach

Brain–machine interface (BMI) technology is one of the favored solutions, as it can decode directly the users’ intentions in terms of their brain signals without nervous peripherals. In 1999, some researchers applied the invasive BMI to train rats to control a robot arm (Chapin et al., 1999). In 2011, Kim et al. (2011) used the microelectrode array signals to control a point-and-click cursor, which made it possible for patients with tetraplegic to use the computer. Later, as a representative work, Hochberg et al. (2012) proposed an invasive BMI technology based on the microelectrode array signals, allowing two patients with long-standing tetraplegia to control a robotic arm for drinking. Even though the invasive BMI is a good solution, it needs an operation on users in advance. The patients may suffer from the expensive craniotomy and additional risks, such as infections and side effects from operations. Therefore, atraumatic non-invasive BMI technology is a better choice for most people.

As Onose et al. pointed out, EEG is the only realistically practical non-invasive BMI approach at present among the existing non-invasive BMI technologies, because it is relatively affordable and easy to set-up (Onose et al., 2012; Ferracuti et al., 2013; Li et al., 2013, 2016; Yu et al., 2013). Other non-invasive BMI technologies, such as functional magnetic resonance imagery, magneto-encephalography, and positron emission tomography, are quite expensive and not portable in terms of the size and electrical energy usage (Onose et al., 2012). Therefore, a number of EEG-based BMI paradigms and systems are exploited and developed in recent years (Schröer et al., 2015; Wang et al., 2015). Active/voluntary paradigm (e.g., Motor imagery, for short as MI) and passive paradigm (e.g., P300 and steady-state evoked potentials, for short as SSVEP) are two basic strategies for the interaction between users and computers. Although some researchers employed the MI-BMI to control a robot arm to perform a task of picking and placing (Wang et al., 2015), the disadvantages are inherent and difficult to accept, such as less control options, more preliminary training, low accuracy, and instability (Li and Yu, 2015). By contrast, P300 evoked potential is more suitable to detect users’ intention. It has been verified that the P300 allows very high accuracy and more optional orders with little training time (less than 5 min), which is essential in practical applications (Prezmarcos et al., 2011; Li and Yu, 2015). In addition, P300-BMI systems do not require subjects to learn how to modulate their EEG, and the P300-BMI was about two times faster than the equivalent Mi-BMI systems (Prezmarcos et al., 2011). A comparison research between P300-BMI system and SSVEP-BMI system has also proved that P300-BMI is more robust for subjects, though SSVEP-BMI has higher bit rate (Lijing et al., 2012). Moreover, the SSVEP-BMI needs to flash consistently in real time to obtain the corresponding signals, which is more tiresome for users. Considering the safety, robustness and less burden, P300-BMI system is more suitable and applied in the ID-SIR system.

In order to improve the accuracy and information transmission rate, efficient classification algorithms are necessary. Among numerous P300-BMI applications, support vector machine (SVM) and linear discriminant analysis (LDA) have been used to achieve acceptable results (Lenhardt et al., 2008; Schröer et al., 2015; Simbolon et al., 2015). As pointed out in Lenhardt et al. (2008), compared with other complex classifiers such as SVM, LDA was capable due to its good classification performance as well as low computational and training requirements. Hoffmann et al. successfully applied the LDA to obtain high classification accuracies and bit rates for severely disabled subjects (Hoffmann et al., 2008). Different from most existing LDA-P300 systems with a fixed training-round number (Townsend et al., 2010; Akram et al., 2015; Chang et al., 2016), a self-adaptive Bayesian linear discriminant analysis algorithm is exploited in this paper to classify the P300 signals to obtain the user’s intention. It can effectively decrease the cost of recognition time. The user’s intention is then translated into control commands that are used to control the robot manipulator to execute desired tasks.

### 1.2 Robot Control

For the severely disabled patients, the less brain burden the system brought in, the better patients may feel. The designed intention-driven semi-autonomous intelligent robotic (ID-SIR) system seeks to decrease the need for user continuously sending commands through “shared control” to realize it. Here, shared control means that it is a semi-autonomous robot, which only needs very limited high-level commands of users. It indicates that users do not need to continuously send instructions to the BMI system. In practical applications, the user only needs to send one command to “tell” the BMI block which object is desired. All the other work will be finished automatically by the robot.

### 1.3 Object Perception

Object perception is realized by embedding with the computer vision. Considering the complexity of objects in home/hospital environments, a region-growing algorithm, and a deep convolutional neural network (CNN) are implemented in the system for cup detection, as well as a depth information based vision localization technology is exploited and applied. Compared with the state-of-art system with color-based classifier (Schröer et al., 2015), the deep CNN method is more powerful and accurate. For instance, robot in Schröer et al. (2015) can only grasp a very specific cup in a predefined place, but the proposed ID-SIR system can grasp any learnt object from any initial position in the range of vision and robot attainability.

Before ending this section, the main contributions of this paper lie as below.

- A non-invasion type intention-driven semi-autonomous intelligent robotic (ID-SIR) system is designed to assist severely disabled users for drinking. To the best of authors’ knowledge, it is the first time to realize a non-invasion type mind controlled robot to grasp a desired object in a random place and deliver it to the user’s mouth.
- A novel depth camera-based visual detection and localization method is employed in the perception layer of the proposed ID-SIR system, which can recognize and locate the desired beverage container in any place in the range of visual and robot reachable regions and the user’s month.
- A self-adaptive Bayesian linear discriminant analysis algorithm is applied to the proposed ID-SIR system, which can effectively decrease the cost of recognition time.
- Experiments and user studies are presented to verify the effectiveness, robustness, and high accuracy of the proposed ID-SIR system.

The remainder of this paper is organized in four sections. Section 2 presents the whole system in detail. The methods used in the ID-SIR system are stated in Section 3. The experiment results are discussed in Section 4. Section 5 concludes this paper with final remarks.

## 2 System Overview

**Figure 1:**
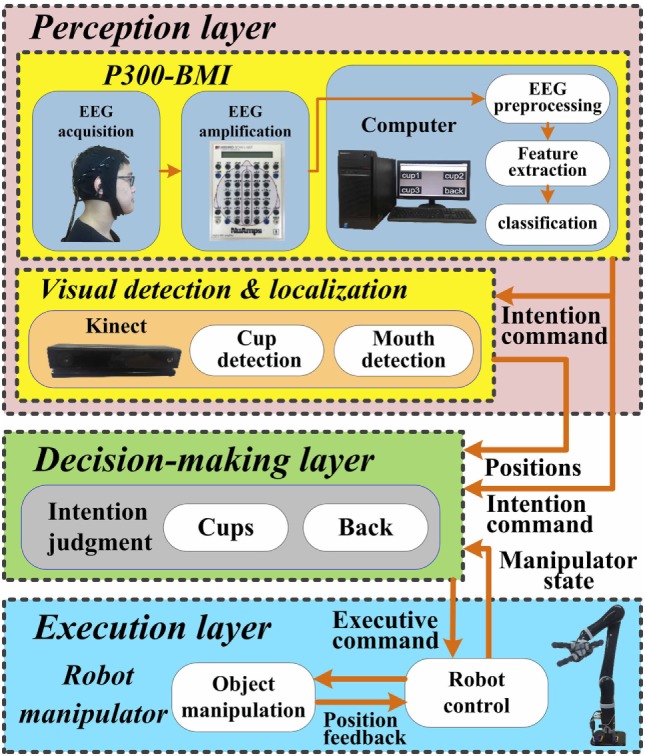
System architecture of the ID-SIR system.

In this section, the working mechanism and information transmission process of the proposed ID-SIR system (as shown in Figure 1) is stated in detail. From Figure 1, we can see that the ID-SIR system includes triple layers, i.e., the perception layer, decision-making layer, and execution layer. The perception layer of the system includes a P300-based brain–machine interface subsystem and a visual detection and localization subsystem. The decision-making layer is about how to convert and transmit the intention of users to the control commands of robots. The execution layer is used for robot control.

First, in the BMI subsystem, an EEG cap and a direct-current amplifier (NuAmps) are applied to acquire brain signals. After preprocessing of the signals and feature extraction, a self-adaptive Bayesian linear discriminant analysis (SA-BLDA) algorithm is employed for classification, and the intention of the user is obtained. Finally, an intention command is sent to the decision-making layer and the visual detection and localization subsystem as an output signal.

Second, in the visual detection and localization subsystem, two Microsoft Kinects are applied as the vision input sensors. With the help of region growing algorithm and deep neural network, the positions of the beverage containers are detected and obtained. Applying the Kinect software development kit (SDK), the position information of the user’s mouth is detected. The position information of the desired beverage container and the user’s mouth are then sent to the decision-making layer in real time.

Third, the decision-making layer works as a connector and coordinator between the other modules, which is responsible for information transition and decision-making. It should decide when and how to deliver which beverage container to the mouth of the user according to the inputs from perception layers and feedback from the execution layer.

Fourth, in order to grasp the desired beverage container and deliver it to the mouth flexibly, a robot manipulator of six degrees-of-freedom (DOF) with three fingers (KINOVA JACO^2^ robot manipulator) is applied. Through motion planning and control, the executive commands, generated by the decision-making layer, are well preformed on the robot manipulator to move along the expected path and finish the drinking task.

## 3 Methods

In this section, the algorithms and working mechanism of three layers in the ID-SIR system are presented in detail. Specifically, it includes perception layer (including BMI and computer vision), decision-making layer, and execution layer.

### 3.1 Brain–Machine Interface

In this section, the BMI subsystem of the proposed ID-SIR system is stated in detail. Specifically, it includes data acquisition and amplification, graphical user interface, time series and control mechanism, and mapping intentions to execution commands.

#### 3.1.1 Data Acquisition and Amplification

First of all, EEG cap is worn by the user and the software setting is prepared. With the application of the cap, the scalp signals referenced to the right ear are detected.

**Figure 2:**
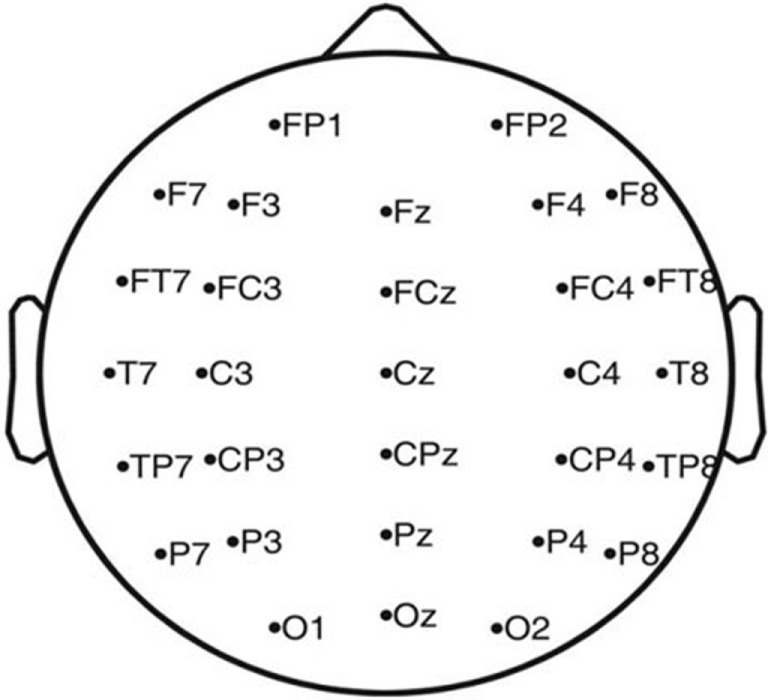
Distribution of the 30 electrodes (expect referenced electrode and ground electrode).

In the experiment, a 32-channel Quik-CapTM (from Compumedics, Neuroscan, Inc.) is employed. The horizontal electrooculograph (HEOG) and vertical electrooculograph (VEOG) are about eye movements that are not necessary in our data analysis process. Therefore, the two channels are ignored in the designed BCI of the ID-SIR system. The corresponding names of electrodes and distribution of remaining 30 channels are shown in Figure 2. As the P300 signals are mainly produced in parietal lobe and occipital lobe, most of the sampling electrodes distribute in these zones.

Second, the captured EEG signals from the cap is amplified, recoded, and transmitted to the computer by a NuAmps device (Compumedics). In the signal acquisition process, all the impedances of the electrodes should be less than 5 KΩ, the sampling rate of the signals is 250 Hz, and the output band pass of the NuAmps device is between 0.5 and 100 Hz.

#### 3.1.2 Experiment GUI Design

**Figure 3:**
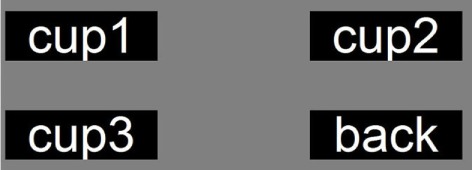
GUI of the proposed ID-SIR system.

In order to attain the user’s intention to control the robot manipulator to deliver the object (such as a bottle or a cup) to the month of the user, a P300-based speller system with 4 symbols are designed (see Figure 3). It is displayed as a 2 × 2 matrices, and each button is attached with a white word and black background in idle periods. As can be seen from Figure 3, the 4 symbol buttons of the GUI are “cup1,” “cup2,” “cup3,” and “back.” Here, the number of symbols denotes the intention which can drive the robot to grasp the *i*th object (cup/bottle) and deliver it to the user’s mouth (i = 1, 2, and 3). Symbol button “back” denotes the intention that drives the robot to put the object back.

#### 3.1.3 Time Series and Control Mechanism

**Figure 4:**
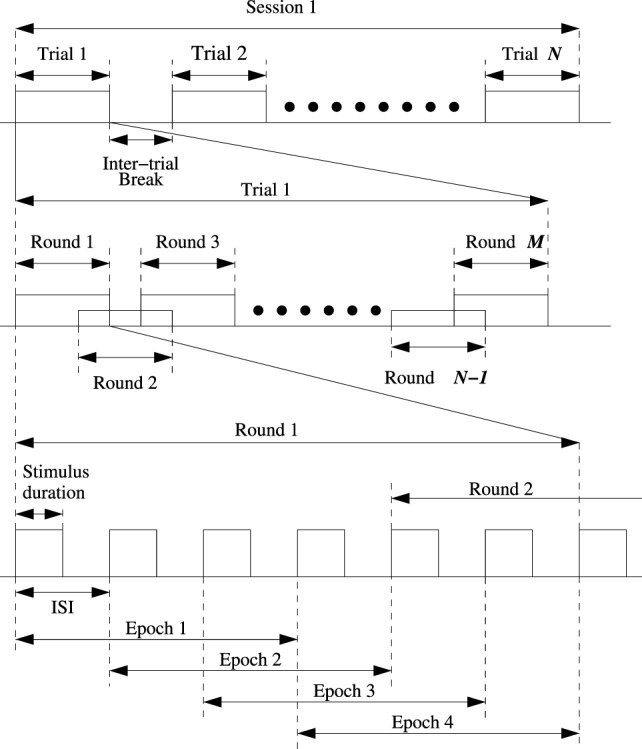
Time series chart of one session: *M* trials per session and *N* rounds per trail.

In the proposed ID-SIR system, the user’s intention is recognized by a self-adaptive P300-based BMI system that works with the time series shown in Figure 4. When the button flashes, it changes into green background and black words.

A session is a user’s off-line training or online testing time period, i.e., a subject’s time cost in the training/testing experiment. In the training process, a character training time is a trial; and in the testing process, a character recognition time is a trial.

In the proposed ID-SIR system, one session includes *N* trials and each trail corresponds to the recognition time cost of a symbol button. Moreover, one trial is divided into *M* rounds. The number of rounds *M* is a self-adaption value determined by the user’s mental state. The time period of a round spans from the flash of the first button to the recover of the final button. The corresponding time is denoted by *t*_round_. In general, the more rounds it takes, the more accurate the recognition will be and the more time the system will spend.

In order to enhance the efficiency, a small number of rounds are expected if the accuracy is satisfied to some extent. According to the actual applications, a trial is set as 10 rounds in the proposed ID-SIR system. Each of the four buttons flashes only once per round, and the total time cost of a round is *t*_round_ = 1.2 s. The stimulus duration is the time cost when one button keeps continuous lighting. In this system, the stimulus duration is 100 ms. Moreover, it is not necessary to start one button’s flashing after others finish. The delay time between one button’s flash and another button’s staring point is called inter-stimulus interval (ISI). The ISI is 200 ms in the ID-SIR system. An epoch is the time period within which P300 signal is recorded and detected. In other words, the P300 signal can be found in an epoch if the user pays attention to the flash button during the corresponding epoch. In the ID-SIR system, *t*_epoch_ = 600 ms.

#### 3.1.4 Mapping Intentions to Executive Commands

In order to accurately map the intentions to task commands, a GUI and a decision-making block are necessary. As can be seen from Figure 3, there are four executive commands totally, i.e., “cup1,” “cup2,” “cup3,” and “back.” During the task execution, the flashing button stimulates the eyes, and the P300 signals are detected, recognized, and converted to executive commands. The robot manipulator is driven by the executive commands to deliver the expected cup to the user’s month.

To do so, a self-adaptive Bayesian linear discriminant analysis (SA-BLDA) algorithm is exploited. In this self-adaptive algorithm, the round number *M* is dynamically and automatically determined on the basis of the user’s mind state and the quality of the signals. The presented SA-BLDA algorithm considers both of the accuracy and the recognition speed.

##### 3.1.4.1 BLDA Algorithm Description

To recognize the acquired P300 signals, a Bayesian linear discriminant analysis (BLDA) is exploited in the proposed ID-SIR system. Considering a regularization parameter, the BLDA algorithm can avoid overfitting problem (Hoffmann et al., 2008).

(a) Assume that a training set (***x***, ***t***) consists of *P* sampling points, denoted by vector ***x*** ∈ *R^P^*, and ***x*** = (*x*_1_, ⋯ , *x_P_*)^T^. Since we need to estimate whether it is a P300 signal or not, it is a logical problem, and thus target value ***t*** ∈ {−1, 1}.

For Bayesian regression theory, target values ***t*** consists of ***x*** linearly weighted by ***w*** with Gaussian noise ***n***_*noise*_ as bellow.

(1) $$\text{t}={\text{w}}^{\text{T}}\text{x}+{\text{n}}_{\mathrm{noise}}.$$

The uncertainty over the value of the target variable can be described by using a Gaussian probability distribution. That is to say, ***t*** has a Gaussian distribution with the mean *μ* = ***w***^T^***x***, and the variance *σ*^2^ = *β*^−1^, i.e.,

(2) $$p\left(\text{t}|\text{x},\text{w},\beta \right)=N\;\left(\text{t}|\mu ,\sigma^{2}\right)=N\;\left(\text{t}|{\text{w}}^{\text{T}}\text{x},\beta^{-1}\right)\;\;,$$

where parameter *β* is the reciprocal of the variance, which denotes the precision of the Gaussian probability distribution.

For the convenience of analysis, we suppose the P300 signals of all the trials are independently and identically distributed. If the number of training samples is denoted by *Q*, for *Q* independent experiment samples and *P* total sampling numbers, inputs ***X*** can be denoted as $\text{X}=\left\{x_{1},x_{2},\cdots ,x_{Q}\right\}\in R^{P\times Q}$. Considering the number of functional keys *K*, number of trails *N*, and number of rounds *M*, the experiment samples *Q* = *N* *⋅* *M* *⋅* *K*. If *C*_Channels_ channels are used, and the sampling number of a section selected P300 signal is denoted by *S*_Samples_, the total sampling number *P* = *C*_Channels_*⋅S*_Samples_. According to the definition of a joint probability, the joint probability of independent experiment samples is determined by the product of the marginal probabilities for each sample value separately. Therefore, the likelihood function is

(3) $$\begin{array}{l} p\left(\text{t}|\text{X},\text{w},\beta \right)=\prod_{n=1}^{Q}N\;\left(t_{n}|{\text{w}}^{\text{T}}{\text{x}}_{n},\beta^{-1}\right)\;,\\ \;={\left(\frac{\beta }{2\pi }\right)}^{Q∕2}\cdot \text{exp}\;\left(-\frac{\beta ∥{\text{X}}^{\text{T}}\text{w}-\text{t}{∥}^{2}}{2}\right). \end{array}$$

(b) For utilizing the Bayesian framework and for the convenience of analysis, a prior distribution over the polynomial coefficients ***w*** is considered. For simplicity, a zero mean Gaussian distribution is formulated as

(4) $$\begin{array}{l} p\left(\text{w}|\alpha \right)=N\;\left(\text{w}|0,\alpha^{-1}\text{I}\right)\;\\ \;={\left(\frac{\alpha }{2\pi }\right)}^{\frac{P+1}{2}}\left(\frac{\epsilon }{2\pi }\right)\;\exp\;\left(-\frac{\alpha }{2}{\text{w}}^{\text{T}}\text{I}\prime \left(\alpha \right)\text{w}\right)\;, \end{array}$$

where parameter *α* decides the precision of this Gaussian distribution. For the linear regression with *P*th order polynomial, the total element number of feature vector ***w*** is *P* + 1. In practical applications, parameter *ε* is usually a small value. Matrix ***I*** is a unit matrix, and ***I***′(*α*) is

$$\text{I}\prime \left(\alpha \right)=\left[\begin{array}{cccc} \alpha & 0 & \cdots & 0\\ 0 & \alpha & \cdots & 0\\ \vdots & \vdots & \ddots & \vdots \\ 0 & 0 & \cdots & \epsilon \end{array}\right]\;\;.$$

Based on Bayes theorem (Bishop, 2006), the posterior distribution for ***w*** is

(5) $$p\left(\text{w}|\text{X},\text{t},\alpha ,\beta \right)=\frac{p\left(\text{t}|\text{w},\beta \right)p\left(\text{w}|\alpha \right)}{\int p\left(\text{t}|\text{w},\beta \right)p\left(\text{w}|\alpha \right)d\text{w}}.$$

For simplify, training set {***X***, ***t***} can be replaced by **D**. Equation (3) is reformulated as

(6) $$p\left(\text{D}|\text{w},\beta \right)={\left(\frac{\beta }{2\pi }\right)}^{Q∕2}\cdot \text{exp}\;\left(-\frac{\beta ∥{\text{X}}^{\text{T}}\text{w}-\text{t}{∥}^{2}}{2}\right),$$

and equation (5) can be rewritten as

(7) $$p\left(\text{w}|\text{D},\alpha ,\beta \right)=\frac{p\left(\text{D}|\text{w},\beta \right)p\left(\text{w}|\alpha \right)}{\int p\left(\text{D}|\text{w},\beta \right)p\left(\text{w}|\alpha \right)d\text{w}}.$$

From equation (7), we see that the posterior distribution of ***w*** is proportional to the product of the prior distribution and the likelihood function, i.e.,

(8) p(w|D,α,β)∝p(D|w,β)p(w|α),

where ***w*** can be determined by finding the most probable value of ***w*** given data set {***X***, ***t***}. In equation (8), the likelihood *p*(***D***|***w***, *β*) and prior *p*(***w***|*α*) are computed by equations (6) and (4), respectively. The posterior distribution of ***w*** is Gaussian because both of the prior and likelihood are Gaussian, and the mean *m* and covariance *C* are

(9) $$\begin{array}{l} m=\beta \;{\left(\beta {\text{XX}}^{\text{T}}+\text{I}\prime \left(\alpha \right)\right)}^{-1}\text{Xt}, \end{array}$$

(10) $$\begin{array}{l} C={\left(\beta {\text{XX}}^{T}+\text{I}\prime \left(\alpha \right)\right)}^{-1}, \end{array}$$

where *α* and *β* can be computed by an iterative algorithm (Mackay, 1992).

(c) When a new input sample $\hat{x}$ is obtained, the distribution function of its predictive regression value $\hat{t}$ is

(11) $$p\left(\hat{\text{t}}|\beta ,\alpha ,\hat{\text{x}},\text{D}\right)=\int p\left(\hat{\text{t}}|\beta ,\hat{\text{x}},\text{w}\right)p\left(\text{w}|\beta ,\alpha ,\text{D}\right)d\text{w}.$$

The predictive distribution (11) is also a Gaussian distribution, and the mean and variance are, respectively, as

(12) $$\mu =m^{\text{T}}\hat{\text{x}},\;\sigma^{2}=1∕\beta +{\hat{\text{x}}}^{\text{T}}C\hat{\text{x}}.$$

In this ID-SIR system, the decision is made by mean *μ*.

##### 3.1.4.2 Self-Adaptive Algorithm Design

**Figure 5:**
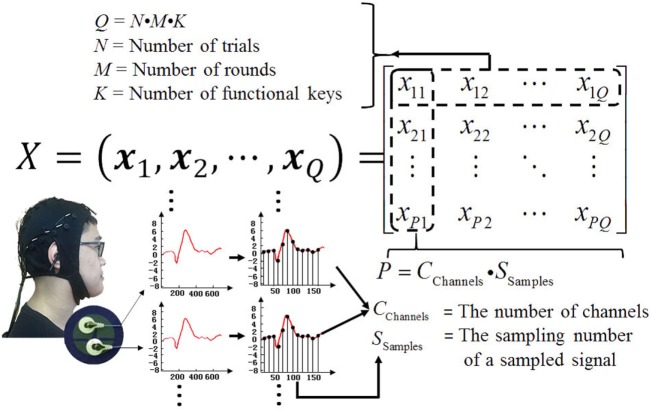
Elements of matrix ***X***. (The individual agrees to publish his photo).

First, during each stimulus period, epoch data need to be preprocessed. Specifically, the sampled EEG data (about 150 discrete points) in 600 ms in each channel are filtered by a narrowband filter with frequency 0.1–20 Hz. In order to compress the data, the narrowband signal data are then sampled again once every 6 points. They are denoted by symbol *S*_Samples_ (see Figure 5). All the 30-channel signals (i.e., *C*_Channels_ = 30) are combined as a new vector ***x*** with *P* = *C*_Channels_*⋅S*_Samples_ dimensions. During online test, 4 functional keys flash per round, and we can get 4-epoch EEG data. It means 4 feature vectors can be obtained at each round.

**Figure 6:**
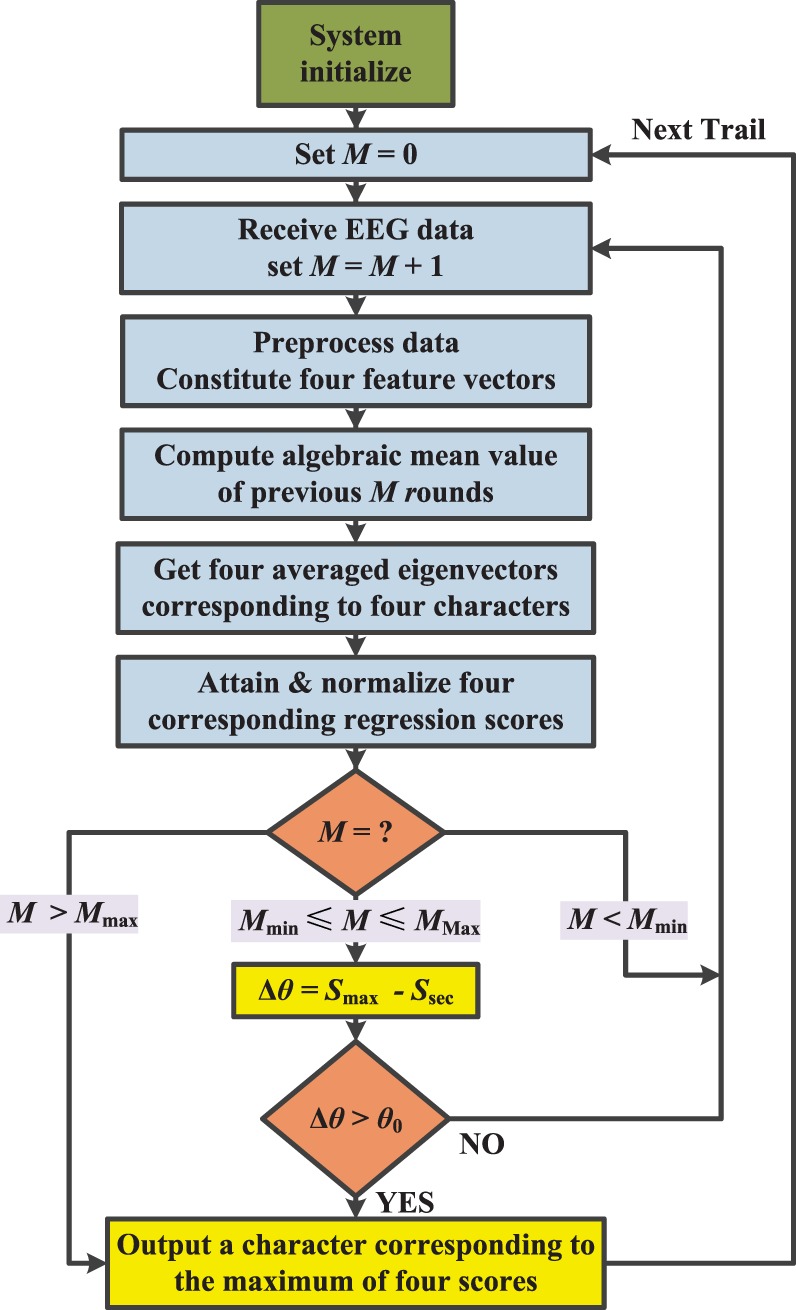
Online classification process of the SA-BLDA Algorithm.

Second, in order to recognize the 4 characters (i.e., “cup1,” “cup2,” “cup3,” and “back”), an SA-BLDA algorithm is exploited, and the corresponding flowchart is shown in Figure 6. When the first P300 signal of one trail comes, the round number *M* is set to zero after the system initializes. When a new EEG signal including 4-epoch data of a new round comes, the round number *M* is set to be *M* + 1. Afterward, all the EEG signal data of 4 epochs at the *M*th round are preprocessed and 4 feature vectors are constituted (each vector includes 30 channels data). The algebraic mean value of the previous *M* rounds feature vectors is computed. In the SA-BLDA algorithm, 4 characters are used, so 4 averaged eigenvectors corresponding to the 4 characters are obtained (i.e., each character corresponding to one $\hat{x}$ in (12)). From equation (12), 4 regression scores (i.e., *μ* in equation (12)) can be obtained. These scores are then normalized between 0 and 1, and denoted by notation *S*. Parameters *M*_min_ and *M*_max_ denote the minimum and maximum number of repeated rounds, respectively. In the proposed ID-SIR system, *M*_min_ = 3 and *M*_max_ = 8. Threshold *θ*_0_ is set in view of training results. The specific selection method is described in the next section.

##### 3.1.4.3 Selection of Threshold Parameter *θ*_0_

Selection of threshold parameter *θ*_0_ is a balance issue between classification accuracy and information transfer rate (ITR). A practical system is expected to have high classification accuracy and ITR. To achieve this aim, curves of accuracy and ITR with various *θ*_0_ are firstly presented. In the ID-SIR system, since the ITR drops while *θ*_0_ increases, *θ*_0_ is set at the point where the curve of accuracy first reaches its highest value. A concrete application example is illustrated in Section 4.

### 3.2 Visual Detection and Localization

**Figure 7:**
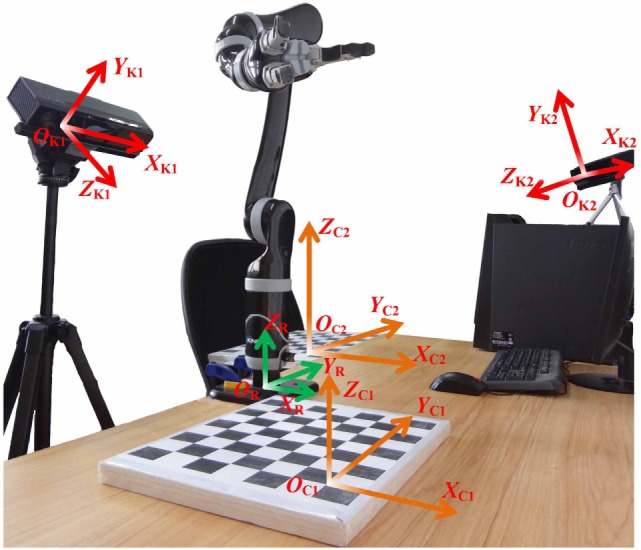
An illustration of coordinate transformation.

In order to realize the automatic task of assistive drinking, it is essential to recognize and locate the desired object as well as the user’s mouth. As shown in Figure 7, two Kinect sensors are applied to execute perception tasks. One is placed in front of the user to detect the position of the user’s mouth, while the other is set up beside the table to recognize and locate the cup, bottle, and pop can. The robot manipulator is placed on one side of the table between Kinect and the user’s chair. In the ID-SIR system, the desired object (such as a cup, a bottle, and a pop) can be put at anywhere in the cross field of Kinect’s scanning zone and robot manipulator’s working region (i.e., the area around the black and white calibration board in Figure 7).

In the ensuing sections, the coordinate transformations from camera coordinate system to the world coordinate system and further to the robot coordinate system are first discussed. The methods of the mouth and object (cup, bottle, and pop can) detection and localization are then analyzed in detail.

#### 3.2.1 Coordinate Transformation

In order to control the robot manipulator to grasp and move an object, the position information of the object in the robot coordinate system needs to be known.

First, camera calibration and transformation from the camera coordinate system to the calibration-board coordinate system are implemented.

The camera coordinate systems of two Kinects (denoted by K1 and K2), calibration-board coordinate systems (denoted by C1 and C2), and robot coordinate system (denoted by R) are shown in Figure 7. The relationship between the camera coordinate system and the calibration-board coordinate system is formulated as

(13) $$\left[\begin{array}{l} X_{\text{K}}\\ Y_{\text{K}}\\ Z_{\text{K}}\\ \;1 \end{array}\right]=\left[\begin{array}{cc} {\;}_{\text{C}}^{\text{K}}R & {\;}_{\text{C}}^{\text{K}}T\\ 0 & 1 \end{array}\right]\;\left[\begin{array}{l} X_{\text{C}}\\ Y_{\text{C}}\\ Z_{\text{C}}\\ \;1 \end{array}\right],$$

where *X*_K_, *Y*_K_, and *Z*_K_ represent the three-dimensional position information in the camera coordinate system of Kinect; *X_C_*, *Y_C_*, and *Z_C_* represent the three-dimensional position information in the calibration-board coordinate system; ${\;}_{C}^{K}R$ and ${\;}_{C}^{K}T$ stand for rotation matrix and translation matrix.

In the ID-SIR system, a common camera calibration method is used to determine intrinsic and extrinsic parameters of Kinect (Zhang, 2000), with which parameters ${\;}_{C}^{K}R$ and ${\;}_{C}^{K}T$ are obtained. By using the SDK of Kinect, the three-dimensional position information of all the points of the object is obtained. The method of getting the three-dimensional position information of the object and mouth in the camera coordinate system will be illustrated in the following sections.

Second, the three-dimensional position information of the object and mouth in the camera coordinate system is transformed into the calibration-board coordinate system as

(14) $${\left[\begin{array}{l} X_{\text{C}}\\ Y_{\text{C}}\\ Z_{\text{C}}\\ \;1 \end{array}\right]}_{\text{ Obect}}={\left[\begin{array}{cc} {\;}_{\text{C}}^{\text{K}}R & {\;}_{\text{C}}^{\text{K}}T\\ 0 & 1 \end{array}\right]}^{-1}{\left[\begin{array}{l} X_{\text{K}}\\ Y_{\text{K}}\\ Z_{\text{K}}\\ \;1 \end{array}\right]}_{\text{ Object}},$$

where ${\;}_{C}^{K}R$ and ${\;}_{C}^{K}T$ are obtained during camera calibration of Kinect.

Third, the three-dimensional position information of the object and mouth in the calibration-board coordinate system is transformed into the robot coordinate system as

(15) $${\left[\begin{array}{l} X_{\text{R}}\\ Y_{\text{R}}\\ Z_{\text{R}}\\ \;1 \end{array}\right]}_{\text{ Obect}}={\left[\begin{array}{cc} {\;}_{\text{C}}^{\text{R}}R & {\;}_{\text{C}}^{\text{R}}T\\ 0 & 1 \end{array}\right]}^{-1}{\left[\begin{array}{l} X_{\text{C}}\\ Y_{\text{C}}\\ Z_{\text{C}}\\ \;1 \end{array}\right]}_{\text{ Object}},$$

where ${\;}_{C}^{R}R$ and ${\;}_{C}^{R}T$ stand for rotation matrix and translation matrix.

Fourth, the three-dimensional position information of the object (cup, bottle, and pop can) and mouth in the robot coordinate system are sent to decision-making layer to implement the drink delivering task.

#### 3.2.2 Object Detection and Localization

As mentioned above, a Kinect sensor is employed to collect the three-dimensional point cloud in the camera coordinate system. We first implement a plane extraction algorithm for background detection and elimination. Next, an object segmentation in the non-background proportion of the point cloud is applied. According to the collection of potential objects’ three-dimensional point sets in the camera coordinate system, the corresponding RGB images of potential objects are isolated and identified with the recognition algorithm based on the library which includes images of the target object. After the recognition and coordinate transformation, the three-dimensional position information of the selected potential object in the robot coordinate system is obtained and sent to the decision-making layer to implement robot manipulator control.

##### 3.2.2.1 Background Extraction

In order to recognize and locate the desired object on the table rapidly and accurately, plane extraction for background– foreground separation is essential. In the ID-SIR system, a region growing (RG) algorithm is exploited to search the horizontal background plane *H*_Plane_.

**Figure 8:**
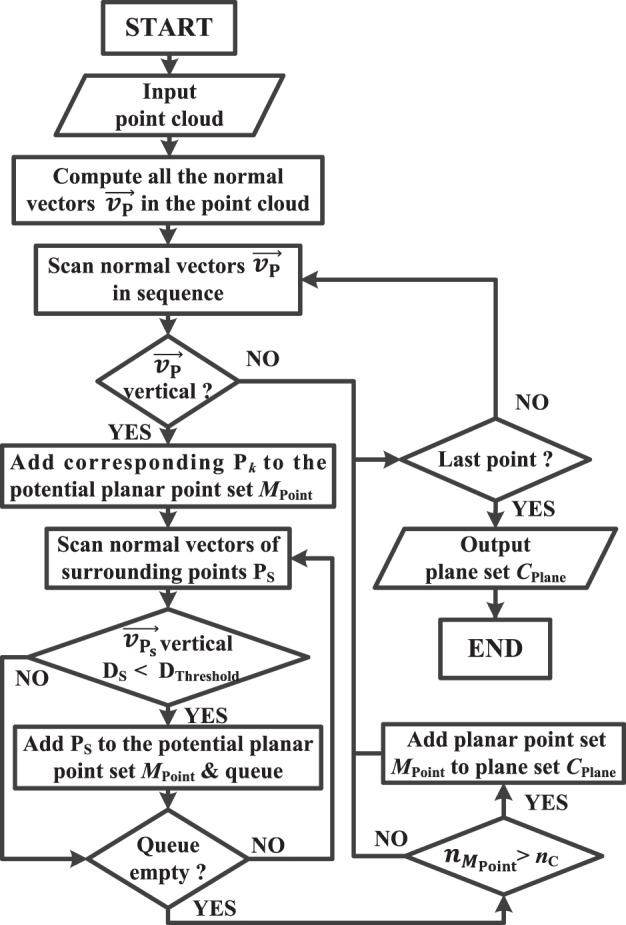
A flowchart describing the procedure of the region growing algorithm.

In the point cloud, we assume that the horizontal plane is a plane where all the normal vectors of points are nearly perpendicular. According to this assumption, all the neighboring points with nearly perpendicular norm vectors are considered as the points on the same horizontal plane. Based on this hypothesis, the RG algorithm is developed, and the corresponding flowchart is shown in Figure 8.

**Figure 9:**
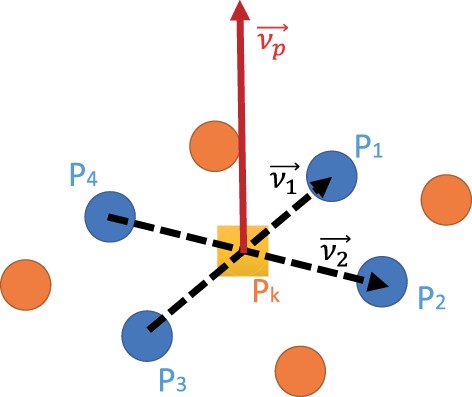
An illustration of normal vector ${\vec{v}}_{p}$ of point P*_k_*.

First of all, the normal vectors of each point in the point cloud are calculated. Without loss of generality, the point P, whose coordinate information is (*x_k_*, *y_k_*, *z_k_*) in the point-cloud space (or termed camera coordinate system), maps to the point of which the pixel coordinate is (*i_k_*, *j_k_*) in the pixel space (or termed image coordinate system). As shown in Figure 9, the normal vector ${\vec{v}}_{\text{P}}$ of point P*_k_* is computed as

(16) $${\vec{v}}_{\text{P}}={\vec{v}}_{1}\times {\vec{v}}_{2},$$

where ${\vec{v}}_{1}={\text{P}}_{1}-{\text{P}}_{3}$ and ${\vec{v}}_{2}={\text{P}}_{2}-{\text{P}}_{4}$, P_1_(*i_k_*, *j_k_*_−1_), P_2_(*i_k_*_+1_, *j_k_*), P_3_(*i_k_*, *j_k_*_+1_), and P_4_(*i_k−_*_1_, *j_k_*) are the four surrounding points beside *P_k_* in the image coordinate system. All the normal vectors of the points in the point cloud are computed according to equation (16).

Second, search all the normal vectors that are nearly parallel to the perpendicular direction and add them into a potential horizontal plane set *M*_Point_. Here, the point P*_k_* can be seen as a seed (i.e., a starting point) of the region growing, in which the four surrounding points P_S_ of the seed are checked whether their normal vectors are perpendicular and the distance D_S_ between P_S_ and the seed are smaller than a threshold value D_Threshold_.

The surrounding qualified points are collected into the potential planar point set *M*_Point_ and inserted into a queue. They work as new seeds of the region growing. The circulation of the region growing will stop only when the queue is empty. Moreover, if the number of potential point set $n_{M_{\text{Point}}}$ is larger than a certain value *n*_C_, the potential planar point set *M*_Point_ would be added into the plane set *C*_Plane_. Finally, when the scanning of all the normal vectors ${\vec{v}}_{\text{P}}$ is completed, the plane set *C*_Plane_ will be output as the horizontal plane *H*_Plane_.

##### 3.2.2.2 Object Segmentation

**Figure 10:**
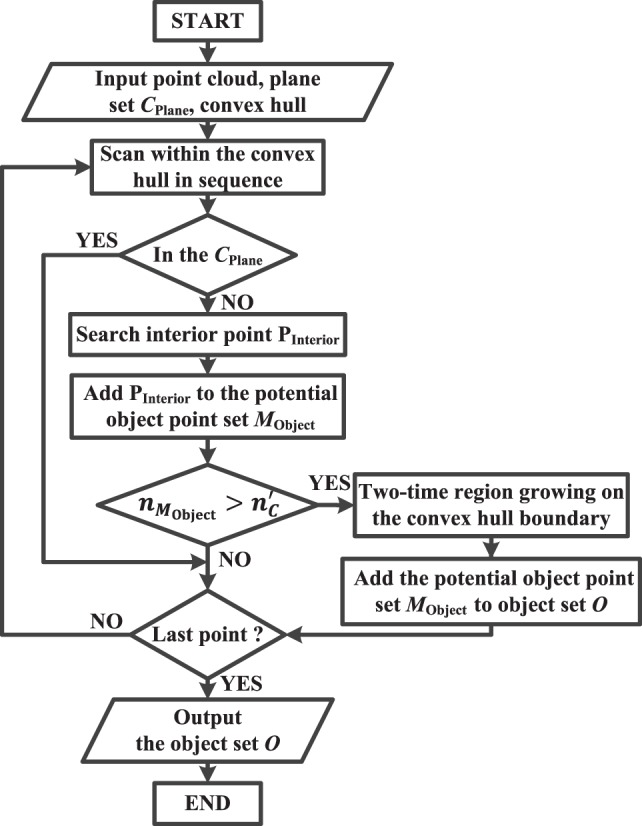
A flowchart describing the object segmentation procedure.

**Figure 11:**
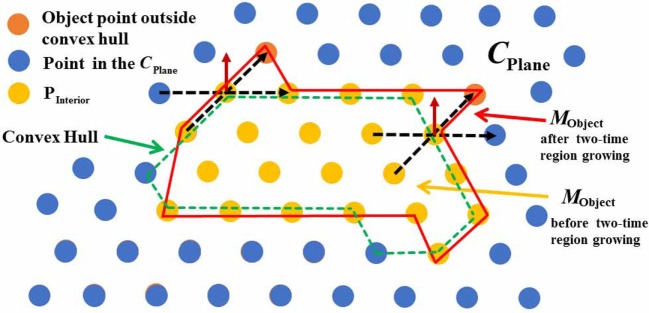
Schematic diagram of two-times region growing algorithm in object segmentation.

In order to segment the expected object from the background, convex hull searching and two-times region growing (RG) algorithms are exploited. The flowchart of the algorithm is shown in Figure 10. The schematic diagram of two-times region growing algorithm is illustrated in Figure 11.

First, according to plane set *C*_Plane_, the convex hulls of objects in the RGB image are computed. A convex hull is the minimum polygon, which roughly describes the outline of an object.

Second, two-times region growing algorithm is proposed to obtain a complete object. The first-time region growing is applied to obtain all the point sets within the convex hulls, and the second-time region growing algorithm is used to handle the convex hull boundary so as to obtain a complete object. Specifically, there are three steps.

- **Step 1.** Traverse the three-dimensional point cloud. Judge whether the points in the point cloud are inside the convex hulls and belong to plane set *C*_Plane_. If the points are inside the convex hulls (i.e., the points inside the green dotted line) but do not belong to the plane set *C*_Plane_, they are considered as interior points P_Interior_ of the object. These interior points will be put into the potential object point set *M*_Object_, and considered as the seed of the region growing.
- **Step 2.** Starting from the seed, if the four points around the seed are inside the convex hulls, but do not belong to plane set *C*_Plane_, and the distance between two points is less than a threshold, then these four points are regarded as interior points P_Interior_ of the object and will be put into the potential object point set *M*_Object_. All the qualified interior points P_Interior_ are collected and put into a potential object point set *M*_Object_. If the number $n_{M_{\text{Object}}}$ of the points in potential object point set *M*_Object_ is larger than a certain value ${n\prime }_{\text{C}}$, the set *M*_Object_ is considered as a real object point set.
- **Step 3.** In order to avoid erroneous judgment of the points near the convex hull boundary, two-times region growing algorithm is exploited to obtain the complete object. In Figure 11, the green dotted line represents the convex hull and the red solid line represents the object region after two-times region growing process. If a point belonging to object point set *M*_Object_ is on the convex hull boundary (i.e., the yellow points on the green dotted line), then the point is considered as a seed of two-times region growing. If any of the four points (i.e., the orange points) surrounding the seed are outside the convex hull and the distance between two points is less than a threshold, then the corresponding points surrounding the seed are considered as the part of the object and put into potential object point set *M*_Object_. In addition, the points surrounding the seed are considered as new seeds as the next round judgment until there are no such points. Finally, all the potential object point sets *M*_Object_ are put into the total object set *O*.

##### 3.2.2.3 Object Recognition

**Figure 12:**
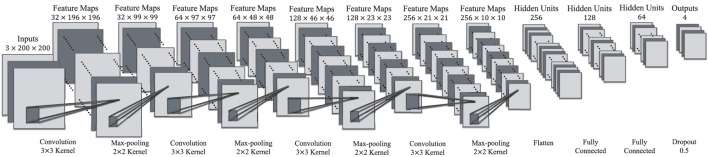
An illustration of the architecture of our CNN.

In order to recognize objects effectively, a deep convolutional neural network (CNN) is designed and applied. Specifically, the architecture of our CNN is presented in Figure 12.

The network contains eight layers with weights: the first four are convolutional layers and the remaining are fully connected layers. Every convolutional layer is followed by a max-pooling layer with kernels of size 2 × 2. The neurons in the fully connected layers are linked to all neurons in the previous layer. The rectified linear units (Relu) is applied to every convolutional layer and fully connected layer as the activation function.

The first convolutional layer filters the 3 × 200 × 200 input image with 32 kernels of size 3 × 3 × 3. The second convolutional layer takes the max-pooled output of the first convolutional layer and filters it with 64 kernels of size 32 × 3 × 3. The third convolutional layer has 128 kernels of size 64 × 3 × 3 connected to the max-pooled output of the second convolutional layer. The fourth convolutional layer has 256 kernels of size 128 × 3 × 3. After the convolutional layers, a flatten layer is employed to transformed the multidimensional feature maps into single dimensional feature maps, which can be put into the fully connected layers. Four fully connected layers have 256, 128, 64, and 4 hidden units, respectively. Between the third and forth fully connected layers, “dropout” technique is applied to reduce the overfitting problem by setting the output of each hidden neuron to zero with probability 0.5. The output of the last fully connected layer is connected to a 4-way softmax which produces a distribution over the 4 class labels (i.e., background, cup, bottle, and pop can).

##### 3.2.2.4 Object Location

After the recognition, the position information of the desired beverage container in the camera coordinate system is calculated as the mean value of position information of all the points of the actual object point set. With the coordinate transformation from the camera coordinate system to the robot coordinate system (see Section 3.2), the three-dimensional position information of the desired beverage container in the robot coordinate system is obtained and is sent to the decision-making layer.

##### 3.2.2.5 Mouth Detection and Localization

In order to complete the automatical assistive drinking task, the position information of the user’s mouth is required. As mentioned at the beginning of Section 3.2, a Kinect sensor is put in front of the user and capture the mouth. With the assistance of the Kinect SDK 2.0, the 3D location of the user’s mouth in the camera coordinate system is obtained. By using the coordinate transformation mentioned in Section 3.2.1, the three-dimensional position information of the user’s mouth in the robot coordinate system is obtained and is sent to the decision-making layer.

### 3.3 Robot Manipulator Control

**Figure 13:**
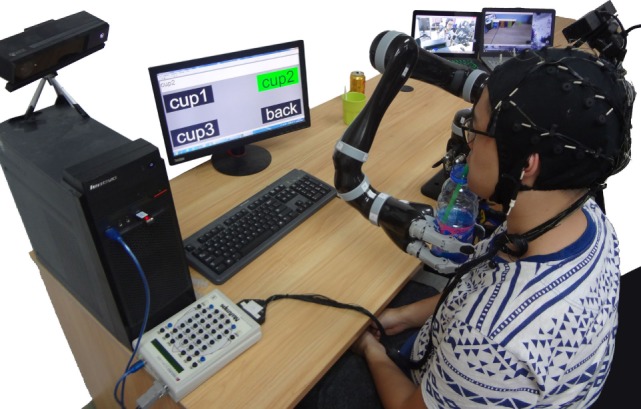
The ID-SIR system assists a user for drinking (the individual agrees to publish his photo).

As shown in Figures 7 and 13, KINOVA JACO^2^ robot manipulator is employed in the ID-SIR system. The robot manipulator has six joints and three fingers. Each finger has a controllable joint and a passive joint. When the controllable joint is grasping an object, the passive joint rotates automatically so that it can hold the object more firmly. Consequently, the robot manipulator has an adequate ability to grasp an object firmly and deliver it to the user’s mouth steadily.

By using the official API, the end-effecter of the robot manipulator (i.e., the three fingers) can be controlled to move from an initial position to an expected position automatically and smoothly. Therefore, only several separated key points in the task space are required to obtain the continuous tracking trajectories of joint space. At present, only the positions of the desired beverage container and the user’s mouth are variable. The remaining position points in the delivering process are predefined. With the consideration of the manipulator’s stability and user’s safety during the task execution, joint velocities of the manipulator are limited at an appropriate speed. Moreover, the manipular state, including position and direction information, is captured and transferred to the robot controller in real time so as to perform accurate control. The manipular state is also sent back to the decision-making layer to make sure that the task is finished.

## 4 Experiments and User Study

The study was approved by the Ethics Committee of South China University of Technology. Written informed consent was obtained from each subject. In order to verify the effectiveness of the proposed ID-SIR system, two experiments are designed: one is the CNN training and the other is whole system evaluation. Moreover, comparisons among existing BMI-based assistive robotic systems and our ID-SIR system are also presented. Figure 13 shows a scenario of a user drinking with the help of the ID-SIR system.

### 4.1 CNN Training

In order to train our CNN to recognize the desired object, a specific data set needs to be established. Without loss of generality, we task three kinds of objects (i.e., a cup, a bottle, and a pop) as an example. The data set was designed to contain 4 classes, i.e., cup, bottle, pop can, and background. Thus, 26,564 images in total, approximately 6,500 samples for each class, were gathered through a Kinect applying the region growing algorithm. The data set was then divided into training set and validation set randomly with a rate of 7:3.

Before training, data augmentation was implemented as generating new images with rescaling and horizontal reflections to reduce the overfitting problem. After 5 epoch of training with “adam” optimization scheme, our CNN finally achieved 0.9905 accuracy on the validation set.

### 4.2 Whole System Evaluation

Eight volunteers were asked to attend the evaluation experiment. The whole system evaluation process consisted of two parts: off-line training and online testing. These volunteers were all healthy subjects (19–21 years old), among which only one subject (i.e., subject 8) had experience in using P300-based BMI system before and the other seven subjects had no experience in BMI system.

#### 4.2.1 Off-Line Training

The EEG signal data were acquired by the following three steps. First of all, a target symbol was given randomly by the computer and displayed in the text box above the four buttons. Second, the subject was asked to pay attention to the given target symbol. Third, the buttons flashed in a random order. Each subject had to complete 40 off-line trials (i.e., *N* = 40) and the chain of potential signals, including useful EEG P300 signals and noises from 30 channels, were recorded in this training process.

After the data acquisition, the data set was processed by the method of self-adaptive Bayesian linear discriminant analysis (SA-BLDA) illustrated in Section 3.1, and the classifier model of the subject was obtained, which was employed to detect the intention command in online testing process.

Figure 15 illustrates the relationship among accuracy, ITR and *θ*_0_ in the SA-BLDA algorithm. As analyzed in Section 3.1.3, parameter *θ*_0_ is set at the point where the curve of accuracy first reaches its highest value. According to this rule, the final selections of *θ*_0_ of all the subjects and the corresponding accuracy and ITR are listed in Table 1. From the table, we can see that the off-line training process is fast, and the accuracy is high. Specifically, all the accuracies are greater than 95%, and all the ITR are less than 20 bits/min.

**Table 1:** Selections of *θ*_0_ and the corresponding accuracy and ITR of eight subjects during off-line training process.

Subject	*θ*_0_	Accuracy (%)	ITR (bits/min)
S1	0.70	98.57	14.83
S2	0.25	100.00	31.00
S3	0.50	95.71	19.31
S4	0.55	100.00	19.60
S5	0.55	97.14	20.30
S6	0.70	98.57	15.31
S7	0.00	100.00	33.33
S8	0.50	95.71	19.84

#### 4.2.2 Online Testing

**Figure 14:**
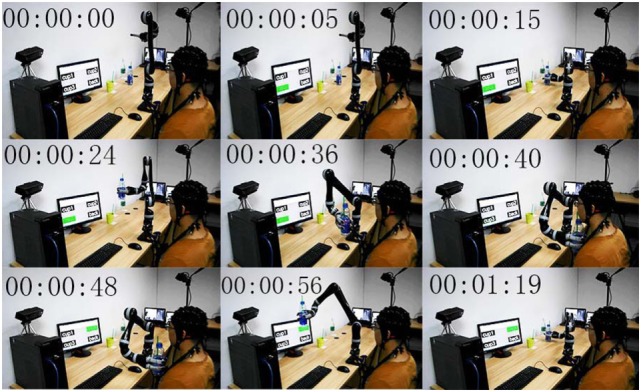
Snapshots of a subject completing one delivering task by using the ID-SIR system (The individual agrees to publish his photo).

During the online testing process, each subject was asked to control the robot manipulator to finish 10 times assistive drinking tasks. In each task, the subject chose a beverage container through the P300-based BMI subsystem and controlled the robot to deliver the beverage container to his mouth, and then sent the “back” command to drive the robot manipulator to send the drink back to its original position. Evidently, two commands were required to complete each task: (i) grasp and deliver the desired beverage container to the month, (ii) put the beverage container back. Therefore, during the online testing experiment, each subject was asked to finish 20 control commands (i.e., trial number *N* = 20). The snapshots of a subject experiencing one assistive drinking task are shown in Figure 14.

**Figure 15:**
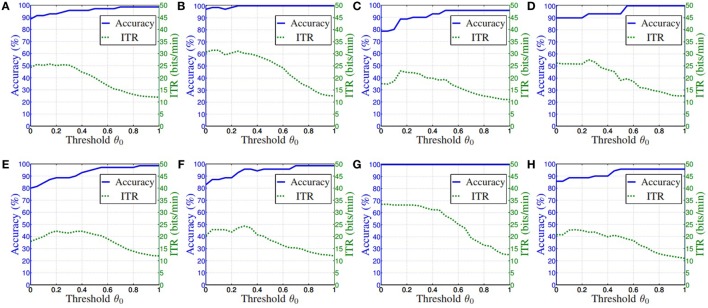
The relationship among accuracy, ITR and *θ*_0_. **(A)–(H)** Subjects 1–8.

The experimental results of eight subjects’ online testing are shown in Tables 2 and 3. In the second and third columns of Table 2, the average round number *M*_a_ and the corresponding average time of P300 signal recognition *t*_P300_ of each subject are presented, respectively. The fourth and fifth columns list eight average time and average accuracy when users completed 10 times drinking tasks. It is worth pointing out that a drinking task includes delivering process and returning process. In other words, the time cost of a drinking task includes time periods of P300 signal recognition, object recognition, object localization, and robot operating. As seen from Table 3 that the mean time of P300 signal recognition is 5.25 s and the average time of completing one task is 84 s in the online testing. The average accuracy of 10 times drinking tasks controlling the robot manipulator is 97.50%. The eight online experiments verify the effectiveness of the proposed ID-SIR system.

**Table 2:** Results of ten times online assistive drinking testing.

Subjects	*M*_a_	*t*_P300_ (s)	*t*_Task_ (s)	Accuracy
S1	7.60	8	86	100%
S2	4.20	4	79	90%
S3	6.10	5	84	100%
S4	6.10	5	87	100%
S5	6.60	5	89	100%
S6	6.95	6	88	100%
S7	4.00	3	82	100%
S8	6.75	6	82	90%
Mean	6.04 ± 1.29	5.25 ± 1.49	84 ± 3	97.50% ± 4.63

**Table 3:** Evaluation of eight subjects in experiments.

Questions	S1	S2	S3	S4	S5	S6	S7	S8	Mean
(Q1) The ID-SIR system can decode your intention precisely (1 = Strongly disagree, 5 = Strongly agree)	5	4	4	4	5	4	3	5	4.25 ± 0.71
(Q2) The ID-SIR system can recognize and localize the desired beverage container in real time (1 = Strongly disagree, 5 = Strongly agree)	5	5	4	5	5	4	5	5	4.75 ± 0.46
(Q3) The ID-SIR system can deliver the desired beverage container to your mouth accurately (1 = Strongly disagree, 5 = Strongly agree)	3	4	4	4	5	5	3	5	4.13 ± 0.83
(Q4) Do you think that the ID-SIR system successfully delivered you the desired beverage container automatically? (1 = Not at all, 5 = Very much)	5	5	5	4	5	5	4	5	4.75 ± 0.46
(Q5) During the experience were you fatigued? (1 = Very much, 5 = Not at all)	4	4	4	4	3	3	3	3	3.50 ± 0.53
(Q6) Do you think that it was a joyful experience? (1 = Not at all, 5 = Very much)	4	4	5	4	5	4	4	4	4.25 ± 0.46
(Q7) Do you think that the ID-SIR system is able to help people with stroke or neurodegenerative diseases to have a drink on their own? (1 = Not at all, 5 = Very much)	4	3	5	3	4	5	5	4	4.13 ± 0.83

Table 3 shows the evaluation of the eight subjects to the proposed ID-SIR system after their experiences. The first four questions are about the functions of the ID-SIR system and the average scores are 4.25, 4.75, 4.13, and 4.75, respectively. These four high scores demonstrate well that the ID-SIR system is very capable and suitable for the assistive drinking tasks. The scores of Q5 and Q6 (reaching to 3.5 and 4.25, respectively) shows that subjects did not bear so much burden in the experiment and the user experience of the ID-SIR system is acceptable. The 4.13 score of the last question indicates that it is possible for the ID-SIR system to continue to perform experiments on patients with stroke and neurodegenerative diseases.

### 4.3 Comparisons with the Existing Systems

In order to highlight the advantages and effectiveness of our system, comparisons among existing BMI-based assistive robotic systems and the ID-SIR system are shown in Table 4.

**Table 4:** Comparisons among existing BMI-based assistive robotic systems and our ID-SIR system.

System	Year	BMI	Visual system	Task	Run time	Accuracy
Hochberg et al. (2012)	2012	Invasive	Experiment judgment	Reach and grasp foam ball	About 7 s	95.6% (Touch)
		MI				62.2% (Grasp)
				Grasp, deliver and drink	≥85 s	67.7%
Katyal et al. (2013)	2013	Non-invasive	Object localization (ECE, SC)	Reach and grasp balls	10.8 ± 0.54 s	Unknown
HARMONIE		SSVEP	Eye tracking (API)			
Wang et al. (2015)	2015	Non-invasive	Object localization (ECE)	Pick and place	97.8 s	100.0% (*Task*)
		MI	Eye tracking (HMM)			47.6% (EEG)
Schröer et al. (2015)	2015	Non-invasive	Object localization (color)	Grasp, deliver and drink	2 min	100.0% (*Task*)
		MI	Mouth detection (haarcascade)			
ID-SIR	2017	Non-invasive	Object localization (RG, CNN)	Grasp, deliver and drink	84 s	97.50% (*Task*)
		P300	Mouth detection (SDK)			

As shown in Table 4, a robotic system in Hochberg et al. (2012) first applied the invasive MI-based BMI technology with a robot manipulator to complete foam balls reaching and grasping tasks and achieved the accuracy as 95.6% (touch) and 62.2% (grasp) spending about 7 s per task. Later, a female patient with tetraplegia and anarthria was assisted by the system to drink coffee from a bottle speeding more than 85 s each time with 67.7% accuracy. However, this system is inefficient and cause great burden on users. Users have to concentrate continually to control the robot manipulator in real time. Besides, sensors need to be implanted in users’ brains and more than 1 month is required for the operation recovery and training. The robotic assistive systems in Wang et al. (2015) and (Katyal et al., 2013) employed non-invasive BMI technology and eye-tracking technology to a control robot manipulator to grasp or pick objects. Besides, vision algorithms, such as Euclidean clustering extraction (ECE) algorithm or sample consensus (SC) algorithm, were also used to locate objects in RGB-D images. However, they did not consider about detection or assistive drinking problems. Regarding the assistive drinking problem, the system in Schröer et al. (2015) incorporated non-invasive MI-based BMI technology with object localization and mouth detection to control the robot. However, the system took almost 2 min to complete one task and the color-based classifier for recognizing a specific colorful plastic cup limited the choices for users.

In order to overcome the deficits of existing systems listed in Table 4, our ID-ARR system applies non-invasive P3000-based BMI technology to complete the assistive drinking task automatically and reduce great burdens on users. It only requires users to have short time training at the beginning and concentrate only two times to give out commands during each whole drinking process. Besides, two-times region growing algorithm and convoluted neural network are applied to recognize and locate the object, which are more effective and generalizable in practical environments.

## 5 Conclusion

In this paper, an intention-driven semi-autonomous intelligent robotic (ID-SIR) system has been designed. The system is composed of a P300-based brain–computer interface (BMI) subsystem, a robot manipulator and an automatic-visual-inspection subsystem. It can detect a desired object and deliver it to the mouth of the user. In order to detect the intention of the user, a self-adaption Bayesian linear discriminant analysis algorithm has been exploited and performed to improve training efficiency and accuracy. Besides, a novel two-times region growing algorithm has been proposed to obtain the complete object. One of the important contributions of this paper is that the combination of BMI and semi-autonomous robot technologies eases the burden on the brain and satisfy user’s assisted-living requirement. By using our system, eight subjects successfully complete 10 times assistive drinking tasks with satisfactory accuracies (≥97.5%). The experiment results have verified the capability of the proposed ID-SIR system and the corresponding algorithms. Compared with the existing BMI system, the advantages of the proposed ID-SIR system are that (1) the object is not predefined and can be put at anywhere in the cross field of sensor’s scanning zone and robot manipulator’s region and (2) both the accuracy and efficiency are considered in the P300-BMI subsystem. Further studies will be conducted to set up the system on a mobile platform and investigate the practical performance on patients.

## Author Contributions

All authors listed have made a substantial, direct, and intellectual contribution to the work and approved it for publication.

## Conflict of Interest Statement

The authors declare that the research was conducted in the absence of any commercial or financial relationships that could be construed as a potential conflict of interest.
